# Supplementary material for: Rapid adaptation to a novel pathogen through disease tolerance in a wild songbird
Source: PLoS Pathog. 2023 Jun 9;19(6):e1011408. doi: 10.1371/journal.ppat.1011408 (PMC10287013; doi:10.1371/journal.ppat.1011408)
Supplement: S1 Appendix — (PDF) [file ppat.1011408.s001.pdf]

Appendix: Supplementary Figures, Tables, Methods, and Results

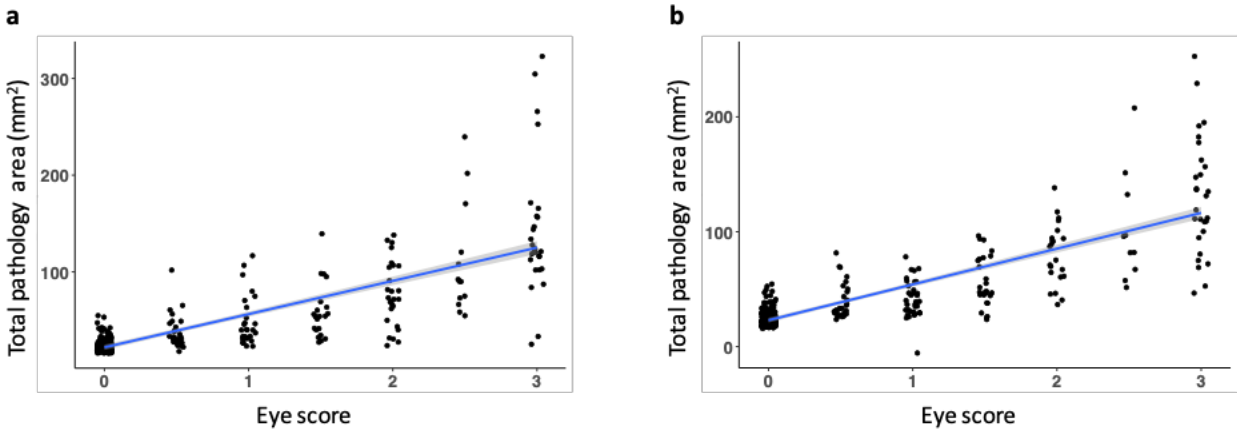

Figure A. Assigned eye scores and the total pathology area were significantly correlated for the (a) left (r_{423} = 0.79, p < 0.001) and (b) right (r_{429} = 0.83, p < 0.001) eyes of finches experimentally infected with MG. Assigned eye scores are on a scale of 0-3, with increments of 0.5. Data points are jittered along the x-axis for better visualization.

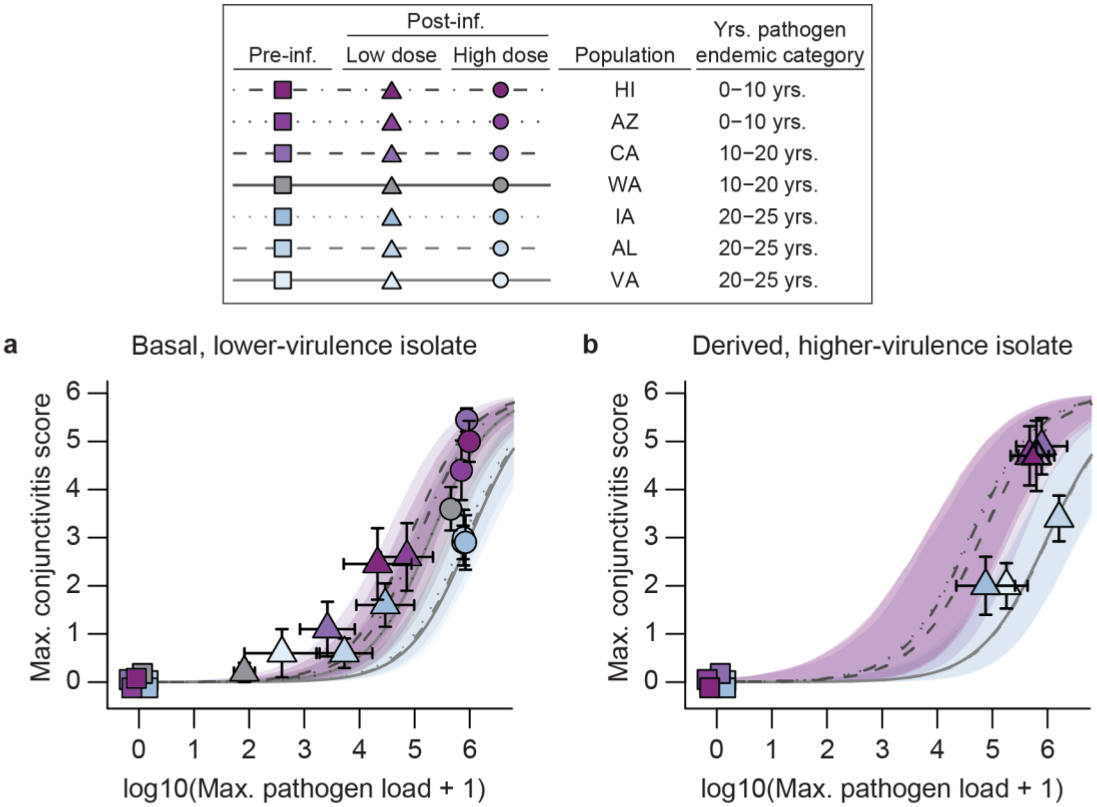

Figure B. Analysis of house finch tolerance of MG by population revealed similar patterns as analysis by category of MG endemism (see main text) using both a (a) lower-virulence and (b) higher-virulence isolate. Of note, the population most intermediate in terms of time since MG arrived, WA, showed intermediate tolerance (a), suggesting that a strict difference between native and non-native populations does not explain our results. Further, among non-native host populations, HI showed less tolerance than IA, AL, and VA, suggesting that the observed differences are better explained by years of pathogen endemism than by native vs. non-native host ranges.

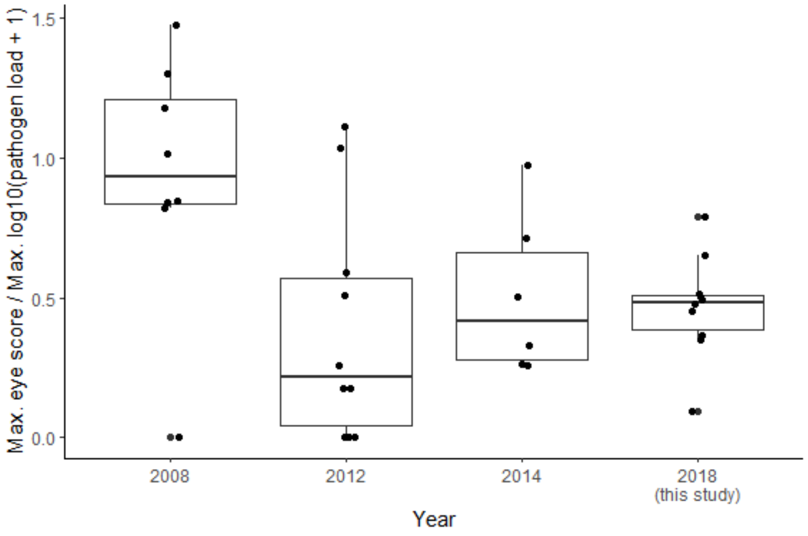

Figure C. A decrease in pathology (eye score) per pathogen load over time suggests that increased tolerance to *M. gallisepticum* infection may have evolved in house finches captured from the same population in Virginia within 15-20yrs of the pathogen's emergence in the early 1990s.

Table A. List of granting agencies and permits granted for this work.

Agency	Permit Number(s)
Alabama Department of Conservation and Natural Resources	9808/9812
Arizona Game and Fish Department	SP624698
California Department of Fish and Wildlife	S-190290001-19044-001
Hawaii Department of Land and Natural Resources	WL19-16
Iowa Department of Natural Resources	SC113
Tennessee Wildlife Resources Agency	2252/33080124
Virginia Department of Wildlife Resources	61440
Washington Department of Fish and Wildlife	19-145a
United States Department of Agriculture	104420/131970/140227
United States Fish and Wildlife Service	MB82600B/MB158404

Table B. List of approximate GPS coordinates for sampling locations where juvenile house finches were collected.

Sampling Location	Approximate GPS coordinates
Blacksburg, Virginia	37.2296° N, 80.4139° W
Ames, Iowa	42.0308° N, 93.6319° W
Tempe, Arizona	33.4255° N, 111.9400° W
Waianae, Oahu, Hawaii	21.4360° N, 158.1849° W
Seattle, Washington	47.6062° N, 122.3321° W
Davis, California	38.5449° N, 121.7405° W
Auburn, Alabama	32.6099° N, 85.4808° W

Table C. Summary of linear mixed effects models ("lmer" function in the lme4 R package) testing for effects of experimental location and year of experiment on log_{10}(max. pathogen load +1) (A) and maximum eye score (B). Both models incorporated population as a random effect with fixed effect of MG isolate, dose, experimental location, and experimental year.

A. log_{10}(maximum pathogen load +1)

Random Effect	Std. Dev.
Population of origin	0.48

Fixed Effects	Satterwhaite's F tests: $F_{num\ df,\ den.\ df}$	Satterwhaite's F tests: *P*	Parameter estimates: estimate	Parameter estimates: t	Parameter estimates: *P*
Intercept	N/A	N/A	3.79	11.94	< 0.0001
Pathogen isolate	$68.1_{1,193.1}$	< 0.0001			
Derived, higher virulence			1.86	8.25	< 0.0001
Pathogen dose	$109.9_{1,190.5}$	< 0.0001			
High			2.25	10.46	< 0.0001
Experimental location	$0.27_{2,195.6}$	0.76			
Univ. of Memphis			0.14	0.35	0.72
Virginia Tech			-0.08	-0.33	0.74
Experimental year	$0.75_{1,8.5}$	0.41			
2019			-0.42	-0.86	0.41

B. Maximum eye score

Random Effect	Std. Dev.
Population of origin	1.01

Fixed Effects	Satterwhaite's F tests: $F_{num\ df,\ den.\ df}$	Satterwhaite's F tests: *P*	Parameter estimates: estimate	Parameter estimates: t	Parameter estimates: *p*
Intercept	N/A	N/A	1.13	1.97	0.09
Pathogen isolate	$47.9_{1,192.4}$	< 0.0001			
Derived, higher virulence			2.12	6.92	< 0.0001
Pathogen dose	$74.5_{1,191.1}$	< 0.0001			
High			2.51	8.63	< 0.0001
Experimental location	$0.67_{2,194.1}$	0.51			
Univ. of Memphis			0.45	0.82	0.42
Virginia Tech			0.36	1.14	0.25
Experimental year	$0.28_{1,7.2}$	0.61			
2019			-0.47	-0.53	0.61

Table D. Comparison of average maximum pathogen load and eye scores broken down by experimental location and year.

A. log_{10}(maximum pathogen load +1)

		Basal, lower-virulence pathogen isolate		Derived, higher-virulence pathogen isolate
Year	Location of experiment	Low dose (mean ± SD)	High dose (mean ± SD)	Low dose (mean ± SD)
2018	Iowa State Univ.	4.72 ± 1.92	6.07 ± 0.24	4.65 ± 1.63
2018	Virginia Tech	3.45 ± 1.90	5.76 ± 0.24	6.02 ± 0.41
2019	Univ. of Memphis	3.06 ± 1.70	5.80 ± 0.23	5.95 ± 1.34
2019	Virginia Tech	2.92 ± 1.08	5.90 ± 0.28	6.21 ± 0.22

B. Maximum eye score

		Basal, lower-virulence pathogen isolate		Derived, higher-virulence pathogen isolate
Year	Location of experiment	Low dose (mean ± SD)	High dose (mean ± SD)	Low dose (mean ± SD)
2018	Iowa State Univ.	2.35 ± 2.31	3.60 ± 1.95	2.08 ± 2.30
2018	Virginia Tech	1.17 ± 1.67	3.76 ± 1.68	4.16 ± 1.95
2019	Univ. of Memphis	0.64 ± 1.22	3.82 ± 1.64	3.54 ± 2.07
2019	Virginia Tech	0.28 ± 0.83	3.79 ± 2.08	4.50 ± 1.54

Table E. Significantly enriched Biological Process Gene Ontology terms for less-tolerant and more-tolerant populations (corrected p-value < 0.05). Genes were separated into upregulated and downregulated gene sets before enrichment analyses were run.

population	Upregulated or downregulated	GO term	GO ID	corrected p-value	Number of genes in term
less-tolerant	upregulated	mitotic cell cycle process	GO:1903047	1.20E-08	20
less-tolerant	upregulated	chromosome segregation	GO:0007059	2.83E-08	15
less-tolerant	upregulated	mitotic cell cycle	GO:0000278	3.94E-08	20
less-tolerant	upregulated	nuclear division	GO:0000280	5.01E-08	15
less-tolerant	upregulated	organelle fission	GO:0048285	1.95E-07	15
less-tolerant	upregulated	cell cycle process	GO:0022402	2.09E-07	22
less-tolerant	upregulated	mitotic nuclear division	GO:0140014	5.70E-07	12
less-tolerant	upregulated	regulation of attachment of spindle microtubules to kinetochore	GO:0051988	7.34E-07	5
less-tolerant	upregulated	leukocyte migration	GO:0050900	8.36E-07	10
less-tolerant	upregulated	mitotic spindle organization	GO:0007052	1E-06	9
less-tolerant	upregulated	cell division	GO:0051301	1E-06	16
less-tolerant	upregulated	antimicrobial humoral response	GO:0019730	1.6E-06	5
less-tolerant	upregulated	sister chromatid segregation	GO:0000819	1.6E-06	11
less-tolerant	upregulated	spindle organization	GO:0007051	1.7E-06	10
less-tolerant	upregulated	response to external stimulus	GO:0009605	2.5E-06	51
less-tolerant	upregulated	nuclear chromosome segregation	GO:0098813	3E-06	12
less-tolerant	upregulated	mitotic sister chromatid segregation	GO:0000070	3.5E-06	10
less-tolerant	upregulated	mitotic spindle midzone assembly	GO:0051256	5.4E-06	4
less-tolerant	upregulated	microtubule cytoskeleton organization involved in mitosis	GO:1902850	7.1E-06	9
less-tolerant	upregulated	mitotic spindle elongation	GO:0000022	8.1E-06	4

less-tolerant	upregulated	attachment of spindle microtubules to kinetochore	GO:0008608	1.2E-05	6
less-tolerant	upregulated	spindle elongation	GO:0051231	1.6E-05	4
less-tolerant	upregulated	spindle midzone assembly	GO:0051255	1.6E-05	4
less-tolerant	upregulated	defense response	GO:0006952	1.7E-05	30
less-tolerant	upregulated	defense response to bacterium	GO:0042742	2.2E-05	8
less-tolerant	upregulated	mitotic cytokinesis	GO:0000281	2.3E-05	6
less-tolerant	upregulated	regulation of cell cycle process	GO:0010564	3E-05	15
less-tolerant	upregulated	cell cycle	GO:0007049	3.1E-05	23
less-tolerant	upregulated	response to bacterium	GO:0009617	3.4E-05	23
less-tolerant	upregulated	positive regulation of leukocyte migration	GO:0002687	3.7E-05	11
less-tolerant	upregulated	leukocyte chemotaxis	GO:0030595	4.6E-05	9
less-tolerant	upregulated	positive regulation of biological process	GO:0048518	4.7E-05	44
less-tolerant	upregulated	response to stress	GO:0006950	0.00011	59
less-tolerant	upregulated	cytoskeleton-dependent cytokinesis	GO:0061640	0.00012	6
less-tolerant	upregulated	locomotion	GO:0040011	0.00014	13
less-tolerant	upregulated	response to biotic stimulus	GO:0009607	0.00016	33
less-tolerant	upregulated	granulocyte chemotaxis	GO:0071621	0.00016	7
less-tolerant	upregulated	humoral immune response	GO:0006959	0.00017	5
less-tolerant	upregulated	regulation of cell motility	GO:2000145	0.00018	25
less-tolerant	upregulated	neutrophil chemotaxis	GO:0030593	0.00021	5
less-tolerant	upregulated	mitotic spindle assembly	GO:0090307	0.00021	6
less-tolerant	upregulated	regulation of cell migration	GO:0030334	0.00022	24
less-tolerant	upregulated	cell motility	GO:0048870	0.00022	19
less-tolerant	upregulated	response to other organism	GO:0051707	0.00029	32
less-tolerant	upregulated	regulation of cell cycle	GO:0051726	0.0003	17
less-tolerant	upregulated	response to external biotic stimulus	GO:0043207	0.00031	32
less-tolerant	upregulated	regulation of leukocyte migration	GO:0002685	0.00032	12

less-tolerant	upregulated	positive regulation of cell cycle	GO:0045787	0.00033	8
less-tolerant	upregulated	tissue development	GO:0009888	0.00035	37
less-tolerant	upregulated	regulation of locomotion	GO:0040012	0.00042	25
less-tolerant	upregulated	cell migration	GO:0016477	0.00044	31
less-tolerant	upregulated	cell chemotaxis	GO:0060326	0.00046	9
less-tolerant	upregulated	cell adhesion	GO:0007155	0.0005	30
less-tolerant	upregulated	positive regulation of cellular process	GO:0048522	0.00053	39
less-tolerant	upregulated	neutrophil migration	GO:1990266	0.00061	5
less-tolerant	upregulated	granulocyte migration	GO:0097530	0.00064	7
less-tolerant	upregulated	biological process involved in interspecies interaction between organisms	GO:0044419	0.00093	33
less-tolerant	upregulated	chromosome organization	GO:0051276	0.00133	13
less-tolerant	upregulated	response to stimulus	GO:0050896	0.00154	99
less-tolerant	upregulated	mononuclear cell migration	GO:0071674	0.00154	6
less-tolerant	upregulated	antibacterial humoral response	GO:0019731	0.00162	3
less-tolerant	upregulated	inflammatory response	GO:0006954	0.00192	13
less-tolerant	upregulated	positive regulation of cell motility	GO:2000147	0.00202	17
less-tolerant	upregulated	cytokinesis	GO:0000910	0.00209	6
less-tolerant	upregulated	microtubule cytoskeleton organization	GO:0000226	0.0022	12
less-tolerant	upregulated	positive regulation of cytokinesis	GO:0032467	0.00233	4
less-tolerant	upregulated	defense response to other organism	GO:0098542	0.00246	21
less-tolerant	upregulated	positive regulation of locomotion	GO:0040017	0.00289	17
less-tolerant	upregulated	response to oxygen-containing compound	GO:1901700	0.00431	27
less-tolerant	upregulated	response to molecule of bacterial origin	GO:0002237	0.00438	13
less-tolerant	upregulated	positive regulation of cell migration	GO:0030335	0.00489	16
less-tolerant	upregulated	spindle assembly	GO:0051225	0.00525	6
less-tolerant	upregulated	positive regulation of response to stimulus	GO:0048584	0.0053	22

less-tolerant	upregulated	regulation of response to stimulus	GO:0048583	0.00601	35
less-tolerant	upregulated	regulation of cell adhesion	GO:0030155	0.00678	13
less-tolerant	upregulated	response to organic substance	GO:0010033	0.00688	42
less-tolerant	upregulated	positive regulation of intracellular signal transduction	GO:1902533	0.00732	14
less-tolerant	upregulated	regulation of mitotic cell cycle	GO:0007346	0.00787	10
less-tolerant	upregulated	regulation of mononuclear cell migration	GO:0071675	0.0079	8
less-tolerant	upregulated	animal organ development	GO:0048513	0.00794	46
less-tolerant	upregulated	immune system process	GO:0002376	0.00833	13
less-tolerant	upregulated	regulation of nuclear division	GO:0051783	0.00894	6
less-tolerant	upregulated	developmental process	GO:0032502	0.00939	77
less-tolerant	upregulated	myeloid leukocyte migration	GO:0097529	0.01012	7
less-tolerant	upregulated	positive regulation of cell cycle process	GO:0090068	0.0103	6
less-tolerant	upregulated	positive regulation of telomere capping	GO:1904355	0.01261	4
less-tolerant	upregulated	positive regulation of signal transduction	GO:0009967	0.0136	17
less-tolerant	upregulated	response to lipopolysaccharide	GO:0032496	0.01379	12
less-tolerant	upregulated	positive regulation of cell division	GO:0051781	0.01577	5
less-tolerant	upregulated	spongiotrophoblast cell proliferation	GO:0060720	0.0162	2
less-tolerant	upregulated	cell proliferation involved in embryonic placenta development	GO:0060722	0.0162	2
less-tolerant	upregulated	spongiotrophoblast layer developmental growth	GO:0090214	0.0162	2
less-tolerant	upregulated	antimicrobial humoral immune response mediated by antimicrobial peptide	GO:0061844	0.01629	5
less-tolerant	upregulated	microtubule-based process	GO:0007017	0.01722	13
less-tolerant	upregulated	positive regulation of leukocyte chemotaxis	GO:0002690	0.01728	7
less-tolerant	upregulated	response to chemical	GO:0042221	0.01775	55
less-tolerant	upregulated	lateral attachment of mitotic spindle microtubules to kinetochore	GO:0099607	0.01947	2

less-tolerant	upregulated	anatomical structure development	GO:0048856	0.01952	66
less-tolerant	upregulated	cytoskeleton organization	GO:0007010	0.02511	17
less-tolerant	upregulated	positive regulation of immune system process	GO:0002684	0.02833	22
less-tolerant	upregulated	response to cytokine	GO:0034097	0.03383	20
less-tolerant	upregulated	positive regulation of signaling	GO:0023056	0.0339	20
less-tolerant	upregulated	positive regulation of cell communication	GO:0010647	0.03448	20
less-tolerant	upregulated	regulation of cellular component organization	GO:0051128	0.03568	22
less-tolerant	upregulated	pteridine-containing compound metabolic process	GO:0042558	0.04288	4
less-tolerant	upregulated	regulation of cytokinesis	GO:0032465	0.04473	4
less-tolerant	upregulated	protein folding in endoplasmic reticulum	GO:0034975	0.0465	3
less-tolerant	upregulated	receptor-mediated endocytosis	GO:0006898	0.04928	9
more-tolerant	upregulated	negative regulation of serine-type endopeptidase activity	GO:1900004	0.00813	2
more-tolerant	upregulated	negative regulation of serine-type peptidase activity	GO:1902572	0.00813	2
more-tolerant	upregulated	mitotic cytokinesis	GO:0000281	0.00964	4
more-tolerant	upregulated	regulation of proteolysis	GO:0030162	0.01373	4
more-tolerant	upregulated	regulation of serine-type endopeptidase activity	GO:1900003	0.01393	2
more-tolerant	upregulated	regulation of serine-type peptidase activity	GO:1902571	0.01393	2
more-tolerant	upregulated	positive regulation of intrinsic apoptotic signaling pathway	GO:2001244	0.01525	2
more-tolerant	upregulated	cytoskeleton-dependent cytokinesis	GO:0061640	0.02921	4
less-tolerant	downregulated	protein localization to cell junction	GO:1902414	0.00346	5

Table F. Comparison of random effects structures in tolerance models from binomial logistic models (generalized linear mixed effects with binomial errors, function "glmer" in the lme4 R package) with eye score as the dependent variable and $\log_{10}$(pathogen load +1), years pathogen endemic, and their interaction as fixed effects.

A. Evolutionarily basal, lower-virulence pathogen isolate

Model	Standard deviation of random effects	Fixed effects	Number of parameters	AICc	ΔAICc
1*	Individual: 0.77	Log_{10}(pathogen load +1) + Yrs. pathogen endemic	5	349.5	0
2	Individual nested in Population: 0.77<br>Population: 1.2×10^{-5}	Log_{10}(pathogen load +1) + Yrs. pathogen endemic	6	351.6	2.1
3	Individual: 0.77	Log_{10}(pathogen load +1) + Yrs. pathogen endemic + Log_{10}(path. load +1) x Yrs. pathogen endemic	7	353.5	4.0
4	Individual nested in Population: 0.77<br>Population: 0.00	Log_{10}(pathogen load +1) + Yrs. pathogen endemic + Log_{10}(path. load +1) x Yrs. pathogen endemic	8	355.6	6.1

B. Evolutionarily derived, higher-virulence pathogen isolate

Model	Standard deviation of random effects	Fixed effects	Number of parameters	AICc	ΔAICc
1*	Individual: 0.92	Log_{10}(pathogen load +1) + Yrs. pathogen endemic	5	171.1	1.3
2	Individual: 0.84	Log_{10}(pathogen load +1) + Yrs. pathogen endemic + Log_{10}(path. load +1) x Yrs. pathogen endemic	7	169.8	0
3	Individual nested in Population: 0.84<br>Population: 3.0×10^{-5}	Log_{10}(pathogen load +1) + Yrs. pathogen endemic + Log_{10}(path. load +1) x Yrs. pathogen endemic	8	172.1	2.3
4	Individual nested in Population: 0.92<br>Population: 4.9×10^{-5}	Log_{10}(pathogen load +1) + Yrs. pathogen endemic	6	173.4	3.6

* Models reported in the main text. Note in B: because models 1 and 2 showed similar support (AICc within 2 units), we report results from the simpler, more parsimonious model, though interpretations about tolerance are the same based on either model.

Table G. Comparison of alternative statistical techniques to assess whether tolerance of infection differs among house finch populations with varying years of *Mycoplasma gallisepticum* endemism. This summary is relevant for models of total eye score using years of pathogen endemism (as a categorical variable) and pathogen load as fixed effects and individual bird as a random effect (see main text). Summaries of fixed and random effects for each model are included in subsequent supplementary tables.

A. Tolerance of infection with evolutionarily basal, lower-virulence pathogen isolate

Model Type (R function, R package)	Range of possible model predictions	AICc	ΔAICc	Interpretation
*Binomial logistic mixed effects model ("glmer", "lme4")	0 – 1 (0 – 6, when rescaled)	349.5	N/A**	With longer pathogen endemism, pathology increases less dramatically with pathogen load (higher tolerance)
Logistic nonlinear mixed effects model ("nlme", "nlme")	0 – 6	703.6	0	" "
Polynomial mixed effects model ("lme, "nlme")	-∞ – ∞	753.7	50.1	" "
Linear mixed effects model ("lme", "nlme")	-∞ – ∞	843.9	140.3	" "

B. Tolerance of infection with evolutionarily derived, higher-virulence pathogen isolate

Model Type (R function, R package)	Range of possible model predictions	AICc	ΔAICc	Interpretation
*Binomial logistic mixed effects model ("glmer", "lme4")	0 – 1 (0 – 6, when rescaled)	169.8	N/A**	With longer pathogen endemism, pathology increases less dramatically with pathogen load (higher tolerance)
Logistic nonlinear mixed effects model ("nlme", "nlme")	0 – 6	327.7	0	" "
Polynomial mixed effects model ("lme, "nlme")	-∞ – ∞	366.0	38.3	" "
Linear mixed effects model ("lme", "nlme")	-∞ – ∞	377.1	49.4	" "

* Models reported in main manuscript. ** because eye score was treated as a binomial variable (see main text) in this model, rather than a continuous variable, AICc comparisons with other models are not valid. Results from all models can be explored by running the R code associated with this manuscript.

Table H. Summary of tolerance models reported in the main text (binomial logistic mixed effects models created using the “glmer” function in the lme4 R package).

**A. Evolutionarily basal, lower-virulence pathogen isolate**					
**Random Effect**	**Std. Dev.**				
Individual	0.77				
	**Likelihood ratio test**		**Parameter estimates**		
**Fixed Effects**	**χ^2**	***p***	**estimate**	**z**	***p***
Intercept	N/A	N/A	-10.85	-8.84	< 0.0001
Log_{10}(max. pathogen load +1)	936.3	< 0.0001	1.83	8.73	< 0.0001
Yrs. endemic	16.39	0.0003			
10-20yrs			1.53	4.66	< 0.0001
None			1.38	4.80	< 0.0001

**B. Evolutionarily derived, higher-virulence pathogen isolate**					
**Random Effects**	**Std. Dev.**				
Individual	0.92				
	**Likelihood ratio test**		**Parameter estimates**		
**Fixed Effects**	**χ^2**	***p***	**estimate**	**z**	***p***
Intercept	N/A	N/A	-9.80	-6.36	< 0.0001
Log_{10}(max. pathogen load +1)	40.34	< 0.0001	1.36	6.35	< 0.0001
Yrs. endemic	26.72	0.0003			
10-20yrs			1.98	3.11	0.002
None			2.31	4.72	< 0.0001

Table I. Pairwise comparison of tolerance estimates between years of pathogen endemism in generalized linear mixed effects models of tolerance. All reflect the Tukey method of p-value adjustment for comparing estimates.

**A. Evolutionarily basal, lower-virulence pathogen isolate**			
**Contrast**	**estimate**	**z**	***p***
20-25yrs – 10-20yrs	-1.53	-4.66	< 0.0001
20-25yrs – 0-10yrs	-1.38	-4.79	< 0.0001
10-20yrs – 0-10yrs	0.15	0.43	0.90

**B. Evolutionarily derived, higher-virulence pathogen isolate**			
**Contrast**	**estimate**	**z**	***p***
20-25yrs – 10-20yrs	-1.98	-3.11	0.005
20-25yrs – 0-10yrs	-2.31	-4.72	< 0.0001
10-20yrs – 0-10yrs	-0.33	-0.47	0.89

Table J. Summary of tolerance models using population, rather than years of pathogen endemism, as a fixed effect (binomial logistic mixed effects models created using the "glmer" function in the lme4 R package).

A. Evolutionarily basal, lower-virulence pathogen isolate

Random Effect	Variance
Individual	0.74

	Likelihood ratio test		Parameter estimates		
**Fixed Effects**	χ^2	*p*	estimate	z	*p*
Intercept	N/A	N/A	-10.81	-8.61	< 0.0001
Log_{10}(max. pathogen load +1)	78.25	< 0.0001	1.80	8.85	< 0.0001
Population	34.97	< 0.0001			
Alabama			0.07	0.15	0.88
Iowa			0.21	0.47	0.64
Washington			1.28	2.56	0.01
California			1.96	3.84	0.0001
Arizona			1.29	2.89	0.04
Hawaii			1.68	3.61	0.003

B. Evolutionarily derived, higher-virulence pathogen isolate

Random Effects	Variance
Individual	0.92

	Likelihood ratio test		Parameter estimates		
**Fixed Effects**	χ^2	*p*	estimate	z	*p*
Intercept	N/A	N/A	-9.81	-6.27	< 0.0001
Log_{10}(max. pathogen load +1)	38.26	< 0.0001	1.63	6.19	< 0.0001
Population	26.77	< 0.0001			
Alabama			-0.03	-0.04	0.96
Iowa			0.01	0.02	0.98
California			1.97	2.66	0.008
Arizona			2.33	3.31	0.001
Hawaii			2.27	3.12	0.002

Supplemental Methods and Results

Comparison of tolerance across time in house finches from Virginia

To provide additional justification for inquiring into the evolution of tolerance in this system, beyond prior two-population comparisons suggestive of host evolution (1-5), we asked if tolerance had changed over time in Virginia, USA, the population of house finches in which *M. gallisepticum* first emerged. To do so, we compared data on pathogen load and pathology (eye scores) from the present study with those from previously published experimental infections of house finches from Virginia (6-8). The earliest of these was performed in 2008, the next in 2012, and the next in 2014. All studies utilized the same isolate of MG, the evolutionarily basal, lower-virulence isolate from the main text, which was derived from a house finch captured in Virginia in 1994 (9). Because prior studies utilized different doses and/or different numbers of exposures, we only retained data from initial exposures at a similar dose to that used in the high dose group in the main text (doses ranged from 1×10^6 – 2.24×10^7 CCU/mL). This represents a total sample size of 34 animals. We also note that prior studies utilized slightly different passages or expansions of the same isolate: Hawley et al. 2010 used the 7th passage, expanded and frozen on 5/24/2004; Adelman et al. 2015 and Leon and Hawley 2017 also used the 7th passage, expanded and frozen on 2/12/2009; and the current experiments used the 6th passage, expanded and frozen on 9/17/2018. As each experiment yielded only a single dose for comparative purposes, we assessed point tolerance, rather than range tolerance (10). That is, we used a single value of pathology / pathogen load, rather than estimating a regression line between the two variables as was possible with multiple doses in the main text. Differences among

experimental years were assessed using a general linear model (ANOVA) in R (11) and post-hoc comparisons, using Tukey adjustments for multiple comparisons, in the emmeans package (12).

House finches from Virginia displayed lower tolerance in the 2008 experiment than in subsequent experiments ($F_{3,30}$ = 4.15, p = 0.01) (Fig. C). Post-hoc comparisons revealed the largest differences between the 2008 and 2012 experiments (t_{30} = 3.3, p = 0.01), followed by the 2008 and 2018 experiments (t_{30} = 2.8, p = 0.04), with all other Tukey-adjusted pairwise comparisons between years showing p > 0.13.

The pattern displayed in Fig. C suggests that tolerance may have changed over time in house finches from Virginia, USA, although it is not conclusive of such an explanation. Nevertheless, it provides justification for exploring the potential evolution of tolerance using a space-for-time substitution, as in the main text. Still, numerous factors, including abiotic or biotic factors that differ in the wild among years could have confounded the earlier results in this supplement. In addition, because these experiments were not originally designed to test differences in host responses over time, the use of different passages and expansions dates of the same isolate could contribute. We note, however, that the expectation of diminished virulence with serial passage (13) was not observed here: the 2008, 2012, and 2014 all used a 7th passage of the isolate, while the experiment described in the main text (performed in 2018) used a 6th passage. But, animals in the 2008 experiment showed the highest levels of pathology per pathogen load, inconsistent with reduced virulence in later passages. Nonetheless, these results

remain merely suggestive of a pattern of evolution, rather than conclusive, pointing to the need for further studies like the one described in the main text.

Tolerance analyzed by population

We analyzed tolerance by population for the lower-virulence isolate experiment, which included animals from Washington (WA), the most intermediate population in terms of time since MG endemism (see Fig. 1 in main text). Linear mixed effects models were constructed as in the main text, except that we replaced pathogen endemism category with population as a fixed effect and used individual, as opposed individual nested within population, as a random effect. Overall, populations with 20-25 years of endemism showed higher tolerance than other populations, as in the main text (Fig. B, Tables C-D;F-J). WA showed intermediate tolerance, which is particularly notable at the high dose, with pathology falling directly in between the more- and less-tolerant populations. In addition, we note that Hawaii (HI), a non-native population, showed a level of tolerance similar to the native populations of California (CA) and Arizona (AZ), rather than other non-native populations of Alabama (AL), Iowa (IA), and Virginia (VA). This suggests that native vs. non-native range alone cannot explain population differences in tolerance.

Quarantine and acclimation procedures

Before transfer to our home institutions (Iowa State University [ISU], University of Memphis [UM], and Virginia Tech [VT]), we housed birds either singly (AZ) or in groups of up to 15 (HI, WA, CA, AL) for up to three weeks (range = 3-20d). After transporting

finches to ISU, UM, or VT, we housed birds singly or in pairs (after quarantine but prior to the start of the experiment, see below) in medium flight cages (76 cm x 46 cm x 46 cm), and provided *ad libitum* food and water daily. On their first day in captivity, birds received only black oil sunflower seeds as food, which transitioned to an 80:20 mix of seed:pellets (Roudybush Maintenance Nibles; Roudybush, Inc., Woodland, CA) for one day, 50:50 seed:pellets for one day, then stabilized at 20:80 seed:pellets for the duration of captivity.

Quarantine and prophylactic medications

Upon arriving at ISU, UM, or VT, we housed birds singly during a two-week quarantine period to ensure all birds were naïve to MG as symptoms of MG develop within two weeks of exposure. On days 3, 7, and 14 of this period, birds were checked for clinical signs of MG infection (i.e., conjunctivitis). After two weeks, we collected blood in heparinized capillary tubes from the brachial vein of each bird. These samples were stored on ice (~4 hours) and then centrifuged for 10 minutes to separate the plasma from red blood cells. We tested plasma from all individuals for anti-MG antibodies (IgY) using a commercially available kit with previously published minor modifications (14) (99-09298, IDEXX, Westbrook, Maine). For our experiments, we used only individuals that showed no evidence of anti-MG antibodies or clinical signs.

To prevent disease from other pathogens present in most house finch populations, we treated birds with several prophylactic medications. Upon arriving at ISU, UM, or VT, we treated all birds with Cankerex (MedPet, Newport, WA, USA) dissolved in drinking water (1 g/L) for five days to minimize risk of trichomoniasis. After this treatment, we then

treated finches with Endocox in drinking water (1.32 g/L; 2.5% toltrazuril, Jedds Bird Supplies, Anaheim, California, USA) for three consecutive days per week to prevent coccidiosis and supplemented birds with probiotics (1 g/L, Bene-Bac Plus bird and reptile supplement, Pet Ag, Inc., Hampshire, IL USA) to promote healthy gut flora for the remaining four days per week. We repeated this treatment regimen for four consecutive weeks and repeated it every two weeks for the duration of captivity. Finally, because malarial parasites can affect the response to MG in house finches (15), for two consecutive days (at least 14 days before experimental infections), we used a micropipette to deliver an oral dose of chloroquine (10 mg/kg/day) and primaquine (day 1: 25 mg/kg, day 2: 15 mg/kg).

Pathogen load quantification

After swabbing conjunctiva, we immediately immersed swabs in 300 µL of TPB five times and wrung out the swab on the side of the tube to transfer MG from the swabs to the solution. We used a new swab for each eye but combined samples from both eyes on each sampling day into a single tube for a given individual. We stored these tubes on ice during sampling periods (~4 hours) and then at -20℃ until extraction. We extracted DNA from swab samples using a Qiagen DNeasy 96 Blood and Tissue kit (Qiagen, Valencia, CA). We performed qPCR assays at a total volume of 15 µL that included 3.525 µL of DNase-free water, 7.5 µL of PrimeTime Gene Expression Master Mix, 0.375 µL or forward and reverse primers, 0.225 µL of probe, and 3 µL of template DNA. Run conditions for the qPCR assay were 3 min. at 95℃ followed by 40 cycles of 3 sec. at 95℃ and 30 sec. at 60℃. On each plate, we included a serially diluted standard curve of a g-Block (Integrated DNA Technologies, Coralville, IA, USA) based on the *mgc*2

amplicon sequence from Grodio et al. (2008) that ranged from 1.81E+01 to 1.81E+09 copies.

Eye pathology (conjunctivitis) measures

We measured the total area of pathology for each individual on days 3, 7, 14, 21, 28, and 34 of infection using ImageJ. To capture pictures of each bird, we held birds in a standardized position (in profile) against a size marker with 1 mm markings. These size markers were used in ImageJ to standardize each picture. We then outlined the pathology for each individual, including all inflammation of the eye and surrounding facial tissues. This area was considered the 'total pathology area'.

Supplement References

1. Adelman JS, Kirkpatrick L, Grodio JL, Hawley DM. House Finch Populations Differ in Early Inflammatory Signaling and Pathogen Tolerance at the Peak of Mycoplasma gallisepticum Infection. The American Naturalist. 2013;181(5):674-89.
2. Bonneaud C, Balenger SL, Russell AF, Zhang J, Hill GE, Edwards SV. Rapid evolution of disease resistance is accompanied by functional changes in gene expression in a wild bird. Proceedings of the National Academy of Sciences. 2011;108(19):7866-71.
3. Bonneaud C, Balenger SL, Zhang J, Edwards SV, Hill GE. Innate immunity and the evolution of resistance to an emerging infectious disease in a wild bird. Molecular ecology. 2012;21(11):2628-39.
4. Bonneaud C, Giraudeau M, Tardy L, Staley M, Hill GE, McGraw KJ. Rapid antagonistic coevolution in an emerging pathogen and its vertebrate host. Current Biology. 2018;28(18):2978-83. e5.
5. Bonneaud C, Tardy L, Giraudeau M, Hill GE, McGraw KJ, Wilson AJ. Evolution of both host resistance and tolerance to an emerging bacterial pathogen. Evolution Letters. 2019;3(5):544-54.
6. Adelman JS, Moore IT, Hawley DM. House finch responses to Mycoplasma gallisepticum infection do not vary with experimentally increased aggression. J Exp Zool A Ecol Genet Physiol. 2015;323(1):39-51.
7. Leon AE, Hawley DM. Host responses to pathogen priming in a natural songbird host. Ecohealth. 2017;14(4):793-804.
8. Hawley D, Dhondt K, Dobson AP, Grodio J, Hochachka W, Ley D, et al. Common garden experiment reveals pathogen isolate but no host genetic diversity effect on the dynamics of an emerging wildlife disease. Journal of evolutionary biology. 2010;23(8):1680-8.
9. Ley DH, Berkhoff JE, McLaren JM. Mycoplasma gallisepticum isolated from house finches (Carpodacus mexicanus) with conjunctivitis. Avian Diseases. 1996;40(2):480-3.
10. Little TJ, Shuker DM, Colegrave N, Day T, Graham AL. The Coevolution of Virulence: Tolerance in Perspective. PLOS Pathogens. 2010;6(9):e1001006.

11. R Core Team. R: A language and environment for statistical computing. R Foundation for Statistical Computing, Vienna, Austria. 2020.
12. Lenth RV. emmeans: Estimated Marginal Means, aka Least-Squares Means. R package version 155-1. 2021.
13. Nicholas R, Ayling R, McAuliffe L. Vaccines for Mycoplasma diseases in animals and man. Journal of comparative pathology. 2009;140(2-3):85-96.
14. Grodio JL, Dhondt KV, O'Connell PH, Schat KA. Detection and quantification of Mycoplasma gallisepticum genome load in conjunctival samples of experimentally infected house finches (Carpodacus mexicanus) using real-time polymerase chain reaction. Avian Pathol. 2008;37(4):385-91.
15. Dhondt AA, Dhondt KV, Nazeri S. Apparent effect of chronic Plasmodium infections on disease severity caused by experimental infections with Mycoplasma gallisepticum in house finches. Int J Parasitol Parasites Wildl. 2017;6(2):49-53.
