# Supplementary material for: Rapid adaptation to a novel pathogen through disease tolerance in a wild songbird
Source: PLoS Pathog. 2023 Jun 9;19(6):e1011408. doi: 10.1371/journal.ppat.1011408 (PMC10287013; doi:10.1371/journal.ppat.1011408)
Supplement: Attachment — Submitted filename: henschen_etal_response.pdf [file ppat.1011408.s005.pdf]

Part I - Summary

Please use this section to discuss strengths/weaknesses of study, novelty/significance, general execution and scholarship.

Reviewer #1: Owing to the spatial extent of the sampling, paired with experimental pathogen challenge, this study represents a rare and important contribution to the areas of ecoimmunology and host evolution in response to novel pathogens. My comments are relatively minor, although I did have some concerns about the statistical analysis, particularly how conjunctivitis score is modeled, the potential importance of reporting random effect variance, and the robustness of the differential expression analyses.

Reviewer #2: This report presents a comprehensive analysis of both the evolution and mechanistic basis of tolerance in the well studied house finch-Mycoplasma system. The main conclusions are that populations with a longer history with Mycoplasma have higher tolerance (and resistance) than populations with a shorter history, and that this is achieved through suppressed immune responses. The study is based on an impressive data set involving common garden experiments with birds from 7 populations. The presentation is clear and the manuscript is well written. The study is a rare example of adaptation in real-time to infectious disease in a wild study system.

Part II – Major Issues: Key Experiments Required for Acceptance

Please use this section to detail the key new experiments or modifications of existing experiments that should be <u>absolutely</u> required to validate study conclusions.

Generally, there should be no more than 3 such required experiments or major modifications for a "Major Revision" recommendation. If more than 3 experiments are necessary to validate the study conclusions, then you are encouraged to recommend "Reject".

Reviewer #1: L325: If conjunctivitis score is the dependent variable, and this ranges from 0-6 (with a maximum of six), have the authors considered using a GLMM instead? A gaussian response (LMM) could theoretically allow your predicted values to extend beyond the maximum eye score possible. A GLMM with binomial response (e.g., cbind(positive eye score, negative eye score)) would bound the model predictions within 0-6. Alternatively, have the authors considered a beta regression model to just model the response as a proportion (e.g., 4/6 eye score)? A poisson response would also be appropriate, although technically that could produce eye scores above 6.

Thank you for the suggestions here—these data are indeed challenging to model while limiting predictions to the 0-6 range. In the previously reviewed version, we had tried an alternative: a non-linear mixed effects model that constrained the response variable between 0 and 6 while allowing for a random effect of population only (not enough data points per individual to do both

in that context). We reported the polynomial model in the original manuscript as it better fit the data than did a linear model, as assessed by AICc.

We have now changed the analysis in the main text (lines 340-399) to the binomial GLMM you suggest, while referring to other options in the supplement, so readers can see that conclusions about the patterns appear to be robust to different statistical approaches. We did not add the poisson GLMM, because, as the reviewer noted, that technique suffers from one of the same problems as the polynomial mixed effects models: predictions above six were possible.

One very important point is that interpreting a logistic curve for tolerance is slightly different than what is normally done with linear or polynomial curves. A right-shift in a logistic curve of pathology (y) vs pathogen load (x) would mean that a group or population is more tolerant. However, such a shift does not require a significant interaction term in the fixed effects, as would be the case of typical tolerance models. Therefore, we note in the methods that with this new modeling framework, we interpret a right-shift as evidence of tolerance (smaller increase in pathology for a given pathogen load) (see lines 385-393).

In the supplement, we also include a table comparing the polynomial fit, a linear mixed model, a logistic (not a binomial) nonlinear mixed effects model, as well as the binomial GLMM suggested by the reviewer (Table S7). While comparison by AICc is not feasible with the latter, since the response variable is different than the former models (see below and main text regarding the dependent variable of GLMM), we think this gives readers the ability to assess the data in multiple ways. The entirety of our data and R code will also be provided so readers can explore each alternative model.

To construct the GLMM, we first needed to adjust our eye scores, as the scale originally included “half” scores, i.e., values of 0, 0.5, 1, 1.5, 2, 2.5, or 3 for each eye, for a total possible of 6. These would not fit the assumptions of a binomial error distribution, so we made the scale coarser, rounding up half scores to the nearest integer to conform to model assumptions. We then created a second “anti” eye score variable, as suggested, calculated as 6 minus total eye score. With these two variables, we modeled the eye score dependent variable as two numbers for each bird: essentially the severity of potential eye pathology achieved (eye_score) and the severity of potential eye pathology not achieved (anti_eye_score). By definition, these two variables always summed to 6 for each individual at each sampling point. We then constructed a generalized linear mixed effects model (GLMM) using the glmer function in the lme4 package in R, with cbind(eye_score, anti_eye_score) as the dependent variable, using fixed effects of max pathogen load and time since endemism with random effects of population and individual, and a binomial error distribution. As noted below, random effects of population were estimated near 0, so we simplified the models, using only a random effect for individual.

L316: The analysis of molecular pathways is slightly vague. Did the authors perform a formal enrichment analysis (e.g., g:Profiler or another similar method)? This would be an ideal method to robustly identify differently up/downregulated pathways.

Thank you for this suggestion. We have performed a formal enrichment analysis using g:Profiler as suggested (methods detailed in lines 324-338; results detailed in lines 515-528). We have

updated Figures 4 and 5, added a supplementary table (Table S5), and included two supplements that provide full detail for the enrichment analyses (Supplement 1) and all differentially expressed genes (Supplement 2).

While our conclusions remain largely the same (i.e., individuals from more-tolerant populations have fewer upregulated immune genes), we agree that this method provides more robust statistical evidence for our conclusions.

L356: Can the authors report variance estimates for the random effects (i.e., individual ID and population)? This could provide important insights into repeatability and population effects.

We have now included updated tables that show estimates of random effects standard deviations (Table S6, S8). In each case, the variance of the random effect of population is minimal, so we report final results from more parsimonious models that include only the random effect for individual.

Figure 2: The fact that the quadratic slope drops below eye score of 0 indicates that the LMM is producing impossible fitted values and why a GLMM with a more appropriate response should be used (e.g., modeling eye score as counts of successes to failures, a proportion, true counts, etc; see above). The same issue is evident in Figure S3.

We have updated the manuscript to include the analysis suggested in the above comment (see Figs. 2 and S2).

L472-479 and Figure 4/5: As it currently reads, the pathway analysis doesn’t demarcate significantly up/downregulated pathways, which should be obtainable with a formal enrichment analysis.

We have performed a formal enrichment analysis using g:Profiler as suggested (methods detailed in lines 324-338; results detailed in lines 515-528).

Reviewer #2: My only major comment is that it is not clear if experiments with all populations were performed at the same time. I guess not, since different populations were captured in different years (L 242-). Would be good with a (supplementary) table specifying which populations were studied in which year, and at which institute. I also wonder if there were any differences in effects of infection (load or symptoms) between institutes and years. I understand that the logistics were necessarily complicated and there is no need for new experiments, but you need to show that the results are not an artefact of non-ideal experimental design (e.g. that experiments with different populations were performed at different institutes or different years, or that time in captivity prior to experiments differed between populations).

We have included this information as two supplementary tables (S3, S4), including new analyses of both pathogen load and clinical signs (eye score) (Table S3). Those analyses are linear mixed effects models and include population of origin as a random effect, while testing

main effects of year, institution, pathogen isolate, and pathogen dose. We did not detect any significant effects of year ($p>0.41$) or institution ($p>0.25$) in any model. We have further clarified these details in the main manuscript (lines 247-249).

Part III – Minor Issues: Editorial and Data Presentation Modifications

Please use this section for editorial suggestions as well as relatively minor modifications of existing data that would enhance clarity.

Reviewer #1: L88: Because MG is defined here, you could move the abbreviation on L90 to here instead.

The text has been changed as suggested.

L133-134: The authors largely focus on using a space-for-time approach, but I think the authors could still better emphasize the importance of the spatial aspects of the study. Multi-site comparisons of immunity in wild hosts are rare and often have low spatial replication (e.g., see a discussion by Becker et al. 2020 JAE). Seven populations sampled across the geographic range of this species is more than most ecoimmunology work and allows the authors to capitalize on a wide range of immunogenetic variation (given the common garden approach).

Thank you very much for this idea. We have incorporated changes in the introduction (lines 134-136) and discussion (lines 577-588) to highlight how we are testing spatial changes in parasite pressure, while controlling for other biotic and abiotic differences. We highlight this as another contribution of the study in the discussion. Indeed, it would be wonderful to have even broader spatial coverage, but we appreciate the recognition that this is a step toward that type of goal.

L142: I think it would make more sense to simply replace “RNAsequencing” with “transcriptomics”

The text has been changed as suggested.

L197: Here and elsewhere, replace “RNASeq” with “RNA-Seq”

The text has been changed as suggested.

L307: “EdgeR” should be “edgeR” to match the R package name.

The text has been changed as suggested.

L385-388 and L389-393: Rather than report the F statistics and p values for each term in the LMM, the authors could consider moving this into an ANOVA table in the main text or supplement and focus on just reporting the significant interactions here (as the main effects are meaningless to interpret with the significant interaction term).

Since we have changed the type of analysis performed to be consistent with earlier suggestions, the main models of tolerance have been simplified and do not include an interaction term. Therefore, reporting in the main text is less cumbersome. We have also included a supplemental table with all model details (Table S8). Notably, the new logistic model still yields the same interpretation: in populations with longer co-evolutionary histories with the pathogen, the increase in pathology with pathogen loads is not as pronounced (higher tolerance).

L396: See the earlier comment about reporting variance estimates for individual and population.

Standard deviation estimates are now reported (Table S6).

Figure 2: It would be nice to see confidence intervals around each of the fitted quadratic slopes here; the authors should consider adding these via bootstrapping or another method for LMMs.

Thank you, we have now included bootstrapped confidence intervals for the fitted glmm suggested in earlier comments.

Figure 3: It would be nice to see confidence intervals around each proportion (e.g., consider using the prevalence package to derive Wilson intervals for smaller sample sizes)

We have added confidence intervals using the Wilson method as suggested. Although these are quite wide, we have also retained the raw data within the figure so readers can easily run the Chi square tests by hand.

L541-545: These are good points; is there any evidence for spatial variation based on these described factors in immune phenotypes (e.g., more classic ecoimmunology markers) in the HOFI system? Additionally, reporting random effect variance for individual and population may be another way to slightly “get at” population effects relative to the fixed effect of interest (years endemic).

Thank you! We do not know of extensive population comparison of more classical ecoimmunology markers, though our groups have begun testing such questions. As to the random effects, we have included the estimates of standard deviation in the random effects in supplemental tables. Of note, the effect of population per se does not seem pronounced in models that incorporate time of MG endemism (Table S6).

L581: Replace “RNAseq” with “RNA-Seq”

The text has been changed as suggested.

L618: One other point the authors may with to briefly discuss is how the expression of immune genes observed here may (or may not!) translate into functional response, given known differences between gene expression and protein abundance. Proteomics may be an applicable approach to mention for future work in this regard (e.g., see Heck & Neely 2020 JPR).

That is a great point! We have updated the text with a discussion of some limitations of transcriptomic studies and the potential usefulness of proteomics, with a focus on potential future work (lines 657-664).

Reviewer #2: Even if there are no differences or effects of timing of experiments or where experiments were performed (see major comment above), the authors should acknowledge that the study is not a proper common garden experiment (which should have involved breeding for at least one generation in captivity to eliminate maternal effects) so that differences between populations could be a result of environmental effects rather than adaptation.

The term ‘common garden approach’ has been replaced with ‘captive experimental approach’, to avoid confusion about the source of birds used in these experiments.

L 72: Ref 5 (Bonneaud) is not about bats. Also, I thought defence against WNS in bats (I assume this is what you have in mind) was a case of tolerance, not resistance.

On line 72, reference 5 refers to Langwig et al. 2017 (Philos. Trans. R. Soc. B) in our main list of references starting on line 701. The Bonneaud reference is number five in our supplemental references. We apologize for the confusion.

While investigation is ongoing, evidence for the evolution of resistance to WNS in bats is stronger than that for the evolution of tolerance (e.g., Langwig et al. 2017, Hoyt et al., 2021 Nature Reviews).

L 100: “of expected”? “Reduce by 40%” better?

In this paper, they are basing population declines on the number of birds expected using historical census data (i.e., Christmas Bird Count data). Thus, all numbers are reported as the number of birds actually seen vs. the number they expected. We have updated the text on lines 99-102 to clarify.

L 268: Briefly describe the function of the Harderian gland, and what organ it corresponds to in mammals (if any).

A brief description has been added as suggested on lines 273-275.

L285: Very deep sequencing, especially considering birds have smaller genomes than for ex mammals!

The smaller genomes do make things easier!

L290: Is there no reference genome for this species?

We are currently developing a reference genome with the Vertebrate Genomes Project, but this genome is not yet assembled and annotated. There is some evidence that *de novo* reconstruction with Trinity is comparable to mapping-first approaches based on reference genomes (Grabherr et al. 2011).

L 420: Asymptomatic means score=0 I assume, but perhaps best to specify?

That is correct and has been clarified in the text on lines 401-402 and lines 460-461.

L 472-: The pathway analysis is a bit unconventional in that you analyze number of DEG in different pathways, rather than which pathways were enriched for DEG; why was the analysis performed in this way?

We have now performed a formal enrichment analysis using g:Profiler (methods detailed in lines 324-338; results detailed in lines 515-528). We have updated Figures 4 and 5, added a supplementary table (Table S5), and included two supplements that provide full detail for the enrichment analyses (Supplement 1) and all differentially expressed genes (Supplement 2).

While our conclusions remain largely the same (i.e., individuals from more-tolerant populations have fewer upregulated immune genes), we agree that this method provides more robust statistical evidence for our conclusions.

L 579: You cannot really say anything about which pathways are suppressed, so delete “less-specific…early”.

The text has been changed as suggested.

L 602-609: Not really meaningful to discuss previous studies in so much detail, so condense this.

We have shortened and condensed this section as suggested (now lines 622-628).
