# Supplementary material for: Rapid adaptation to a novel pathogen through disease tolerance in a wild songbird
Source: PLoS Pathog. 2023 Jun 9;19(6):e1011408. doi: 10.1371/journal.ppat.1011408 (PMC10287013; doi:10.1371/journal.ppat.1011408)
Supplement: Attachment — Submitted filename: Henschen_etal_response.docx [file ppat.1011408.s004.docx]

Manuscript Title: Rapid adaptation to a novel pathogen through disease tolerance in a wild songbird

Revisions made:

1. Added author summary after abstract
2. Removed funding information from manuscript
